# Untapped Resources: Biotechnological Potential of Peptides and Secondary Metabolites in Archaea

**DOI:** 10.1155/2015/282035

**Published:** 2015-10-04

**Authors:** James C. Charlesworth, Brendan P. Burns

**Affiliations:** ^1^School of Biotechnology and Biomolecular Sciences, University of New South Wales, Sydney, NSW 2052, Australia; ^2^Australian Centre for Astrobiology, University of New South Wales, Sydney, NSW 2052, Australia

## Abstract

Archaea are an understudied domain of life often found in “extreme” environments in terms of temperature, salinity, and a range of other factors. Archaeal proteins, such as a wide range of enzymes, have adapted to function under these extreme conditions, providing biotechnology with interesting activities to exploit. In addition to producing structural and enzymatic proteins, archaea also produce a range of small peptide molecules (such as archaeocins) and other novel secondary metabolites such as those putatively involved in cell communication (acyl homoserine lactones), which can be exploited for biotechnological purposes. Due to the wide array of metabolites produced there is a great deal of biotechnological potential from antimicrobials such as diketopiperazines and archaeocins, as well as roles in the cosmetics and food industry. In this review we will discuss the diversity of small molecules, both peptide and nonpeptide, produced by archaea and their potential biotechnological applications.

## 1. Introduction

Archaea are organisms that often thrive in “extreme” environments tolerating conditions that other organisms could not such as a wide range of temperatures from the lakes of Antarctica to the hot springs of Yellowstone, as well as conditions such as high salt of the dead sea or extreme pH conditions of soda lakes in Africa [1–4]. As a result of tolerance to these environmental stressors, proteins from these organisms have been highly valued in biotechnology for their stability and ability to function where other proteins would degrade. However, further research is needed to elucidate, for example, whether metabolites produced by these extremophiles have similar stability and function through chemical modifications such as carboxylation of acyl homoserine lactones discussed below. Enzymes are of particular interest, with many classes being thoroughly investigated such as proteases [5], hydrolases [6], and lipases and esterases [7]. These enzymes have been widely covered in the literature [8, 9], and as such will not be covered in this review.

However, in addition to enzymes already being utilized for biotechnological processes, recent research indicates archaea can produce a wide range of small peptides and secondary metabolites, which could be of considerable interest to biotechnology. Comparatively less is known about biosynthesis in archaea than bacteria; while vitamins and cofactors are readily produced in a number of archaeal strains, there are incomplete biosynthesis pathways possibly due to novel genes being present [10]. The same is true of a number of the metabolites examined in this study despite several having bacterial analogues, and the same genes required for biosynthesis are not present in archaea. Despite a number of recent studies and rise of new technologies such as metabolomics, little is understood regarding the full potential of archaeal metabolites. There are multiple cases of entire metabolite groups being represented at this time by a single archaeal species, most likely due to a lack of study.

In this review we discuss the variety of molecules produced by archaea and their potential role and biosynthesis based on bacterial analogues and how these molecules may be exploited for biotechnological gain.

## 2. Archaeocins

Bacteria have widely produced antimicrobial peptides and proteins termed bacteriocins, which have been utilized in a range of industries. Archaeocins are antibiotic peptides sourced from archaea being found widely amongst haloarchaea (termed halocins) and more recently from the* Sulfolobus* genus (sulfolobicins). Halocin production is thought to be near universal amongst haloarchaea [11], and as a result of this, there is a great deal of diversity within these molecules. Halocins can be divided into two classes based on size: the smaller microhalocins that can be as small as 3.6 kDa to the larger halocins of 35 kDa [12]. The antimicrobial activity of these halocins can also range, with some halocins having narrow range of activity affecting only close relatives, as opposed to a more broadly active A4 halocin capable of inhibiting the growth of* Sulfolobus solfataricus,* a representative of another phylum of archaea [13]. While some bacteriocins are capable of inhibiting archaea [12], there is no confirmed inhibition of bacteria by a halocin, although there have been reports that halophilic archaea are capable of inhibiting halophilic bacteria [14].

One particularly relevant use of halocin producing strains is in the textile industry, which uses considerable amounts of salt in the tanning process. These conditions allow halophiles including some haloarchaea to grow which in turn can damage the product, and halocins have been used to prevent this unwanted growth [15].

Some halocins have also been considered for potential medical use. Halocin H6 has been indicated to have a potential therapeutic benefit in dogs following organ transplant surgery [16]. Halocin production is near universal and relatively few have been fully characterized, in particular their molecular diversity and differences in activity, and it is entirely possible that other therapeutic treatments could result from further study [14]. Another class of archaeocins, Sulfolobicins, were first identified in* Sulfolobus islandicus*, a member of the thermoacidophile group Sulfolobus which grow at low pH range of 2–4 and high temperatures between 65°C and 85°C.* Sulfolobus* species are members of the crenarchaeota phylum as opposed to haloarchaea which are members of the euryarchaeota phylum. Sulfolobicins produced by* S. islandicus *are able to inhibit growth of other closely related strains of* S. islandicus *and other Sulfolobus species. No activity of sulfolobicins against organisms outside of the Sulfolobus phylum has been recorded. Interestingly sulfolobicins appear to be remarkably stable, able to tolerate pH conditions between 3 and 10.7 and as high temperature as 78°C [17]. Sulfolobicins produced by* S. islandicus* appear to be associated with the cytoplasmic membrane and are not generally released into the surrounding medium [18].

## 3. Diketopiperazines

**Figure 1:**
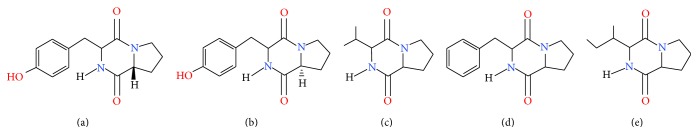
Structure of DKPs known to be produced by the archaeon* H. hispanica*. (a) Cyclo(D-prolyl-L-tyrosine), (b) cyclo(L-prolyl-L-tyrosine), (c) cyclo(L-prolyl-L-valine), (d) cyclo(L-prolyl-L-phenylalanine), and (e) cyclo(L-prolyl-L-isoleucine).

Diketopiperazines (DKPs) also occasionally referred to as cyclic dipeptides have been observed in many bacteria from a wide range of environments and have only recently been identified in a haloarchaeon,* Haloterrigena hispanica* [19]. A schematic illustrating some of the known DKP structures is shown in Figure 1. It is currently unknown how many archaea produce DKPs, with suggestions that* Natronococcus occultus *is also a potential producer as a result of a biosensor assay which can be activated by DKPs or AHLs [19, 20]. DKPs are synthesized in bacteria using one of two pathways, the nonribosomal peptide synthesis (NRPS) or via a novel class of enzymes called cyclic dipeptide synthases (CDPS) [21]. At present there are no functional NRPS gene clusters or CDPS genes identified in archaea. DKPs have a plethora of useful bioactivities that are of potential significance for industrial and medical purposes, including antibacterial, antifungal, and antiviral as well as antitumor activities. Other activities relating to human physiology have also been reviewed [22].

Another interesting activity of DKPs is the ability to activate (and sometimes inhibit) quorum sensing systems in bacteria. Quorum sensing refers to genetic organization in which an organism can control when a phenotype is expressed based on the population levels present, that is, activating a phenotype under a high cell density. Inhibition of these quorum sensing systems in bacteria is thought to be a potential therapy for a range of pathogens such as* Pseudomonas aeruginosa* infections of cystic fibrosis patients [23]. There is also potential for quorum sensing blockers to be used more broadly in industry where biofilms can cause biofouling, a problem for a variety of industries, particularly shipping [24]. Biofilms have also been linked to difficulties in implants and catheters [25], as well as contaminating water pipe systems [26]. DKPs sourced from archaea could potentially be used to block these QS systems in order to prevent these biofilms from occurring.

## 4. Acyl Homoserine Lactones

**Figure 2:**
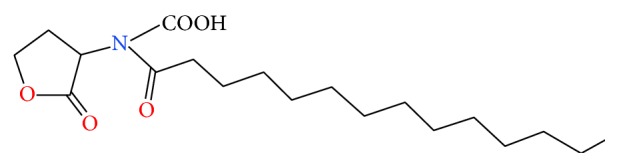
Chemical structure of N-carboxyl-C14-HSL: a putative AHL produced by* Methanosaeta harundinacea*. The carboxylation modification has only been observed in archaea.

Acyl homoserine lactones (AHLs) are well described metabolites controlling a range of quorum sensing phenotypes in bacteria and have recently been identified in archaea. AHLs, while predominantly produced by Gram-negative bacteria, have been shown to interact with eukaryotes as well representing a potential cross-kingdom signaling system [27]. It is important to note the AHLs currently identified in* Methanosaeta harundinacea* have a carboxylation modification previously unseen in bacteria (Figure 2); however, these carboxyl AHLs are still able to activate bacterial biosensors [28]. Short chain AHLs typically degrade quite rapidly under a range of conditions archaea may face such as high temperature and alkaline conditions [29], and thus it is likely that (any) AHL-like molecules in archaea would be either longer chain or possess other characteristics that facilitate stability. However, there is little known what properties the carboxyl modification provides for the molecule in the environment when facing such extreme conditions. Currently the only phenotype known to be AHL regulated in archaea is filament production in* M. harundinacea* [28]. An extracellular protease from extreme haloalkaliphilic* Natronococcus occultus* has also been potentially linked to AHL based quorum sensing but this has not been confirmed [20]. Other phenotypes commonly regulated by quorum sensing in bacteria, however, are present in archaea such as antimicrobial production (see above), cellular competence, and biofilm formation.

AHLs and other quorum sensing molecules are often in control of exopolysaccharide production [30], which could potentially be controlled by quorum sensing. The biofilms of haloarchaea and the extremophiles are of particular interest to biotechnology due to the exopolysaccharides and polyhydroxyalkanoates produced during this particular phenotype. These molecules have many potential uses discussed below.

Many phenotypes governed by quorum sensing in bacteria are of particular interest to bioremediation such as the expression of biofilms, flocculation, and enzyme production. It is possible with a greater understanding of archaeal quorum sensing there could be benefits to bioremediation [28–31]. AHLs are synthesized by AHL synthases, using acyl groups taken from the fatty acid synthesis pathway in bacteria via an acyl-carrier protein ACP [32]. Not only do archaea lack any traditional AHL synthase, they also lack any ACPs [33] as they have very different lipid biochemistry to bacteria, having a fatty acid pathway that may function independently of an ACP type protein. While all known AHL synthases in bacteria are members of the GCN5 acetyltransferase family, it has been suggested that FilI, a histidine kinase, is an AHL synthase in* M. harundinacea* [28].

## 5. Exopolysaccharides and Polyhydroxyalkanoates

Exopolysaccharides (EPS) are high molecular weight carbohydrates produced and released by many different microorganisms including archaea. EPS is thought to provide an organism protection against several environmental insults such as desiccation, predation, or ultraviolet radiation [30]. EPS production in archaea has been primarily researched within the halophile and thermophile groups [34]. EPS molecules have a number of industrial applications such as uses in the food industry as gelling or emulsifying agents. A number of bacterial EPS molecules such as xanthan, curdlan, and dextran are being produced industrially despite a number of these being produced by pathogens such as* Alcaligenes faecalis* [35]. As there are no known archaeal pathogens, it is possible a safer alternative could be found in archaea.

It has been suggested EPS producing* Haloferax* species could be used to remove heavy metal contamination from environments high in salt [36]. The biosorption process of heavy metals by EPS has been documented in other organisms [37]; however, halophilic archaea may be uniquely equipped to survive and produce EPS to reduce contamination in strictly halophilic environments [36].

Another EPS component, polyhydroxyalkanoates, is a water insoluble polymer used as a means of carbon and energy storage in bacteria and archaea. PHAs have received considerable attention in biotechnology as a potential alternative to petrochemical based plastics due to their structural properties and biodegradability [34]. PHAs at present are being produced by recombinant* E. coli* strains; however, there is the suggestion that some halophilic archaea may be ideal producers of PHAs. There are a number of producers of PHAs amongst the haloarchaea [38], the best of which may be* H. medeterrani* with 65% of the dry cell weight being PHA when fed glucose or starch [39]. As* H. medeterrani*  needs 22% salt to grow optimally, there is less of a need for sterile conditions as there is a reduced chance of contamination [40]. The need for high salt also provides an ease of cell lysis by simply changing to fresh water [40]. Current research revolves around improving the production of PHAs through the knockout of nonessential genes, such as those involved in EPS production [41].

## 6. Carotenoids

Carotenoids are naturally occurring pigments that are commonly found in haloarchaea and are responsible for the recognizable reddish pigmentation of the organisms. Carotenoids are widely used as food supplements and colouring agents resulting in an estimated market value of $1.02 billion in 2009 [42]. The role of carotenoids in human health is still being researched; however, some potential benefits have been identified, such as the prevention of chronic diseases and disorders such as cancer [43], chronic heart disease [44], and osteoporosis. There are many carotenoids in nature produced by bacteria, eukarya, and archaea, though few are able to be either chemically synthesized or extracted at a high enough level for industry [42]. Canthaxanthin is a carotenoid used by industry as a feed additive for chickens, fish, and crustacean farms and used in cosmetics. Due to the properties of carnathaxin and its many usages there has been continuing research into producing it in larger quantities. The halophilic archaeon* Haloferax alexandrinus* produces canthaxanthin at a high enough level it could be considered for commercial production of canthaxanthin [45]. It has been suggested by using* H. alexandrinus* that there is less of a concern for aseptic culturing as the higher salt conditions reduce the chance for contamination. As for PHAs described earlier, another benefit to using* H. alexandrinus* to produce carnathaxin is the ease at which cells lyse in fresh water making the carnathaxin easier to extract [45].

## 7. Biosurfactants

Biosurfactants are surfactants produced by a wide range of organisms including archaea and are a mixture of glycolipids, fatty acids, proteins, and sugars. Biosurfactants show many advantages to traditional chemically derived surfactants by being biodegradable, nontoxic, renewable, and active under a range of extreme conditions [46]. Biosurfactants may be able to assist bioremediation of oil spills in soil and water samples, as well as a range of other uses in the food [47], cosmetic, and pharmaceutical industries. Several halophilic archaea have been identified to produce biosurfactants at the highest salt concentration recorded [48]. A species of* Natrialba* was isolated from a solar saltern in Algeria that was found to produce a biosurfactant [49] which was constituted of sugar protein and lipids including rhamnolipids, a class of glycolipid already suggested for use in bioremediation [50] and cosmetics [51]. It was suggested that this* Natrialba* species or possibly other halophilic archaea may be an ideal choice for assisting in the bioremediation of oil spills in saline and hypersaline environments, which can be often contaminated through industrial processes [49].

## 8. Phenazines

**Figure 3:**
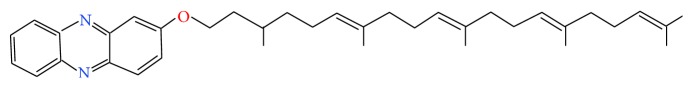
Chemical structure of methanophenazine, a phenazine observed only in archaea.

Phenazines are naturally occurring compounds commonly produced by bacterial genera such as* Pseudomonas* and* Streptomyces* and are known for a variety of biological activities such as antibacterial, antiparasitic, and antitumour effects [52]. While there are a variety of bacterially produced phenazines, there is also an example of a phenazine (methanophenazine) produced by an array of* Methanosarcina* including* Methanosarcina mazei* [53]. An example of such a phenazine structure is shown in Figure 3. This methanophenazine appears to play a key role in the metabolism of the organism as an electron carrier, unlike most other phenazines that are typically considered secondary metabolites [53]. It is unknown whether phenazine production in archaea is limited* to M. mazei* or if it is more widespread. The biosynthesis of phenazines in bacteria typically stems from nonribosomal peptide synthesis, a process responsible for many secondary metabolites and natural products, though as mentioned above there are no known NRPS systems detected in archaea.

## 9. Organic Solutes from Archaea

Organic solutes, also referred to as osmolytes, compatible solutes, and osmoprotectants, are small molecules that assist microorganisms in surviving saline conditions. Organic solutes have several suggested usages in biotechnology as preservatives or cryoprotectants of enzymes and other organic molecules as well as other potential roles such as cosmetics [54]. Organic solutes are widely used by all three kingdoms; dunaliella, an algal species, uses glycine betaine and among bacteria ectoine and glycine betaine are most common [55]. In archaea there are several groups that produce organic solutes such as the halophilic methanogens that produce a range of organic solutes [56]. A novel class of organic solutes, 2-sulfotrehalose, was discovered in the haloalkaphiles such as* N. occultus* [57] and was later found in other related species such as* Natrialba magadii and Halalkalicoccus jeotgali* [58]. Hyperthermophiles also produce a range of compatible solutes considered potentially useful for industry such as trehalose produced by members of the* Sulfolobus* genus which could be used as a cryoprotectant of mammalian cells, as well as having roles in cosmetics and food industries [59]. Other examples such as diglycerol phosphate produced by* Archaeoglobus fulgidus* [60] or di-myo-inositol phosphate from* Pyrococcus furiosus* have been suggested as protein stabilizers under high temperatures.

## 10. Conclusions

While archaea apparently lack traditional gene clusters for natural product biosynthesis such as NRPS, archaea still display a wide diversity of metabolites. Although some metabolites produced by archaea are already in use, others would require significantly more research and development before the applications become apparent and/or economically feasible. Despite this, archaea represent an untapped resource in the field of natural product discovery and could contribute to many areas of industry if fully utilized.
